# A novel anti-proliferative role of HMGA2 in induction of apoptosis through caspase 2 in primary human fibroblast cells

**DOI:** 10.1042/BSR20140112

**Published:** 2015-01-14

**Authors:** Xi Shi, Baoqing Tian, Wenlong Ma, Na Zhang, Yuehua Qiao, Xiaoxue Li, Yu Zhang, Baiqu Huang, Jun Lu

**Affiliations:** *The Institute of Genetics and Cytology, Northeast Normal University, Changchun 130024, China; †The Institute of Audiology and Speech Science of Xuzhou Medical Collage, Xuzhou 221004, China

**Keywords:** apoptosis, caspase 2, HMGA2, WI38 cells, Apaf1, apoptotic protease activating factor 1, caspase, cysteine aspartic acid-specific protease, GFP, green fluorescent protein, HEK-293T cells, HEK-293 cells expressing the large T-antigen of SV40 (simian virus 40), HMGA, high-mobility group AT-hook, HUVEC, human umbilical-vein endothelial cells, MOMP, mitochondrial outer membrane permeabilization, MTT, 3-(4,5-dimethylthiazol-2-yl)-2,5-diphenyl-2H-tetrazolium-bromide, PARP, poly(ADP ribose-polymerase, PI, propidium iodide, SAHF, senescence-associated heterochromatin foci, shRNA, small-hairpin RNA

## Abstract

The HMGA2 (high-mobility group AT-hook) protein has previously been shown as an oncoprotein, whereas ectopic expression of HMGA2 is found to induce growth arrest in primary cells. The precise mechanisms underlying this phenomenon remain to be unravelled. In the present study, we determined that HMGA2 was able to induce apoptosis in WI38 primary human cells. We show that WI38 cells expressing high level of HMGA2 were arrested at G2/M phase and exhibited apoptotic nuclear phenotypes. Meanwhile, the cleaved caspase 3 (cysteine aspartic acid-specific protease 3) was detected 8 days after HMGA2 overexpression. Flow cytometric analysis confirmed that the ratio of cells undergoing apoptosis increased dramatically. Concurrently, other major apoptotic markers were also detected, including the up-regulation of p53, Bax and cleaved caspase 9, down-regulation of Bcl-2; as well as release of cytochrome *c* from the mitochondria. We further demonstrate that the shRNA (small-hairpin RNA)-mediated Apaf1 (apoptotic protease activating factor 1) silencing partially rescued the HMGA2-induced apoptosis, which was accompanied by the decrease of cleaved caspase-3 level and a decline of cell death ratio. Our results also reveal that γH2A was accumulated in nuclei during the HMGA2-induced apoptosis along with the up-regulation of cleaved caspase 2, suggesting that the HMGA2-induced apoptosis was dependent on the pathway of DNA damage. Overall, the present study unravelled a novel function of HMGA2 in induction of apoptosis in human primary cell lines, and provided clues for clarification of the mechanistic action of HMGA2 in addition to its function as an oncoprotein.

## INTRODUCTION

The HMGA2 protein is a member of the HMGA (high-mobility group AT-hook) family, consisting of HMGA1a, HMGA1b and HMGA2 [1,2]. HMGAs are widely expressed in early embryogenesis but are restricted as the fetal development progresses. HMGAs are absent or present at low levels in normal adult somatic cells and tissue [3–5]. As an oncoprotein, HMGA2 is overexpressed in many tumours and plays important roles in stem cell self-renewal, proliferation and differentiation [6]. In contrast to these known functions, however, recent studies have implicated that the HMGA2 protein is specifically accumulated in chromatin in senescent cells, and the ectopic expression of HMGA2 can induce growth arrest in primary cells, followed by the occurrence of senescent phenotypes [7,8] and accumulation of DNA damage [9]. Apparently, more intensive studies are required for further insights into the precise functional mechanisms of HMGA2 protein.

Apoptosis is a delicately orchestrated process that is responsible for many biological functions [10,11]. Apoptosis is initiated by two major pathways, namely the extrinsic (receptor-mediated) and the intrinsic (mitochondria-mediated) pathways [12]. One of the features of apoptotic cell death is the activation of caspases (cysteine aspartic acid-specific proteases), a class of cysteine proteases [13]. Caspases 3 as an effector enzyme [14] is activated through cleavage by the initiator Caspase 9 or -8/10 [15]. Caspase 9 is activated by the Apaf1 (apoptotic protease activating factor 1) apoptosome in the cytoplasm, whose formation is triggered by MOMP (mitochondrial outer membrane permeabilization) and the release of cytochrome *c* from mitochondria to cytosol [16,17]. Caspase-8/10 are activated by the DISC (death-inducing signalling complex) [18,19]. Intriguingly, caspase 2 as one of the most evolutionarily conserved of the caspases [20], exhibits features of both initiator and effector caspases [21,22]. The mechanism of pro-caspase-2 activation in apoptosis remains poorly defined in contrast to other caspases. It was reported that caspase 2 is implicated in cytochrome *c* release and is essential for cytotoxic stress-induced apoptosis in several human cell lines [23–26]. Furthermore, caspase 2 has been increasingly seen as a tumour suppressor, being able to influence many tumour-promoting activities [27–32].

In the present study, we demonstrate that HMGA2 was able to induce apoptosis in primary human cells, a function that has not been previously identified. We also detected the accumulation of DNA damage in HMGA2 expressing cells, which may initialize caspase 2 activation and further induces MOMP to active downstream caspases. Data arising from the present study are important for clarification of the mechanisms of the induction of apoptosis by oncoprotein HMGA2 in primary cells.

## MATERIALS AND METHODS

### Cell culture and reagents

WI38, IMR90 and HEK-293T cells [HEK-293 cells expressing the large T-antigen of SV40 (simian virus 40)] were purchased from the ATCC (USA), and HUVEC (human umbilical-vein endothelial cells) cells were provided by Professor Ju Gu of Peking University. Cells were maintained in MEM (WI38 and IMR90) media and DMEM (Dulbecco's modified Eagle's medium) (293 T) media from Gibco, supplemented with 10% (v/v) FBS (NCD500, Shanghai ExCell Biology Inc for 293T cells. HyClone, USA, Thermo Scientific Inc for WI38 and IMR90). HUVEC cells were maintained in ECM media from ScienCell, supplemented with 100 mg/ml penicillin and 100 mg/ml streptomycin, and kept in a humidified atmosphere containing 5% (v/v) CO_2_ at 37°C.

### Vector construction and viral infection

The pWPXLD lentiviral vectors were used. HMGA2 gene was cloned by RT–PCR from total RNA of senescent WI38 cells. The amplified PCR product was inserted into the PmeI/BamHI or BamHI/EcoRI sites of pWPXLD vector, and then fused with or without EGFP (enhanced green fluorescent protein) gene. Lentiviruses were packed using the HEK-293T cells. Lentivirus supernatant was diluted with culture medium and applied to WI38 cells for 24 h.

### Cell proliferation assay

The MTT [3-(4,5-dimethylthiazol-2-yl)-2,5-diphenyl-2H-tetrazolium-bromide] assay was conducted to measure cell proliferation. WI38 cells stably expressing alien genes transduced by lentivirus were seeded in 96-well plates at a density of about 8000 cells/well. Twenty microliters of MTT (5 mg/ml) was added at 2d–14d after seeding. The samples were incubated at 37°C for 4 h, then the supernatant was discarded, and 100 μl DMSO was added to each well. Absorbance at 492 nm was measured on a microplate reader. Assays were repeated six times, and the survival percentage (%) was calculated relative to the control.

### Western blotting

Western blotting was performed as described previously [43]. The primary antibodies used were: anti-pp53 (1:1,000, CST), anti-p53 (1:1000, CST), anti-p21 (1:500, Santa Cruz), anti-p16 (Santa Cruz, sc-468), anti-caspase 3 (1:1000, CST), anti-PARP [poly(ADP ribose) polymerase] 1 (1:3000, ECTOMICS), anti-HMGA2 (1:5000, ECTOMICS), anti-caspase 9 (1:1000, Bioworld), anti-Bax and anti-Bcl2 (1:1000, CST), anti-caspase 2 (1:1000, KeyGEN), anti-γH2A (1:2000, Millipore) and anti-β-actin (1:10 000, Sungene).

### Immunofluorescence

WI38 cells were grown on coverslips in six-well plates and washed three times with PBS, fixed in 4% (v/v) formaldehyde solution for 10 min and then permeabilized with 0.2% (v/v) Triton X-100 in PBS for 10 min. Cells were blocked with 5% (w/v) BSA in PBS for 1 h at room temperature. Coverslips were incubated with respective primary antibodies for 1 h. The following primary antibodies were used: anti-caspase 3 (1:200, CST), anti-γH2A and anti-cyto-C (1:200, Millipore). The specimens were washed with TBST (TBS containing Tween 20) and incubated for 1 h with TRITC (tetramethylrhodamine β-isothiocyanate)-conjugated secondary antibodies at 1:400 dilutions. Cells were further washed in TBST and DNA was visualized by using DAPI (4′,6-diamidino-2-phenylindole) (1 μg/ml). Images were taken under a confocal laser-scanning microscope (Olympus FV1000).

### Real-time PCR

Total RNA was extracted using a QIAGEN RNeasy Mini Kit (74104), and RT–PCR was performed using a TaKaRa RNA PCR Kit (RR019A). The sequence-specific primers used were indicated as follows. Apaf1 sense: ACATTTCTCACGATGCTACC; antisense: CAATTCATGAAGTGGCAA. CyclinA sense: TTCATTTAGCACTCTACACAGTCACGG; antisense: TTGAGGTAGGTCTGGTGAAGGTCC. CyclinB1 sense: CAGTCAGACCAAAATACCTACTGGGT; antisense: ACACCAACCAGCTGCAGCATCTTCTT. CyclinE sense: GGAAGAGGAAGGCAAACG; antisense: GCAATAATCCGAGGCTTG.

### Annexin-V/PI (propidium iodide) staining

Control and HMGA2-treated cells (0.5×10^6^ cells) were collected by trypsinization. Samples were washed once with PBS and then resuspended in 100 ml Annexin-binding buffer provided by the manufacturer (Alexis). Then 5 ml of Annexin-V–FITC stock solution (Alexis) and 1 mg/ml (final) PI were added and the cells were incubated for 15 min before the stained samples were measured by flow cytometry (Epics XL Beckman coulter). The debris was excluded from analysis. The assay kit was provided by Sungene.

### Cell circle assay

Control and HMGA2 overexpressing cells were centrifuged (300× ***g***, 2 min) and the pellets were resuspended in 1 ml of 70% (v/v) ethanol at −20°C. Cells were fixed at room temperature for 30 min and stored at −20°C overnight. Oligo-nucleosomal DNA fragments were treated by 10 mg/ml RNAse A (Sigma) for 15 min, stained with PI (propidium iodide, Sigma, 5 mg/ml final concentration) for 15 min before measurement. Cells were gated to exclude the debris and then analysed by flow cytometry (Epics XL Beckman coulter).

### Statistical analysis

Data are expressed as mean±S.D.. The statistical significance of differences was assessed by *t* test. In all comparisons, *P<*0.05 (*) was considered statistically significant and *P<*0.01 (**) was considered highly significant.

## RESULTS

### HMGA2-induced growth arrest in WI38 cells

**Figure 1:**
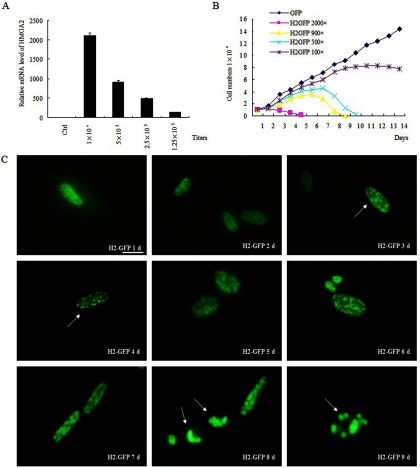
Establishment of the HMGA2-induced cell growth arrest model and the change of heterochromatin (**A**) WI38 cells infected with series dilution of concentrated lentivirus containing HMGA2, in which the relative HMGA2 mRNA level was titred between 100 and 2000 multiples as assessed by qPCR. (**B**) Proliferation of WI38 cells with the indicated expression level of HMGA2 in (**A**) was estimated through cell counting at indicated time. (**C**) Change of heterochromatin foci containing HMGA2-GFP protein in WI38 cells as monitored by fluorescence microscopy at indicated time points after ~500-multiple HMGA2 expression level. The arrows indicate the SAHF-like foci at days 3–4, the apoptotic nuclear change at day 8, and the apoptotic body at day 9. Scale bar: 20 μm.

To establish a model for studying the HMGA2-induced growth arrest in WI38 cells, we ectopically expressed the HMGA2 protein fused with GFP (green fluorescent protein) in WI38 cells using a lentiviral delivery system driven by the strong EF1α promoter. By consulting a previous study with lung cancer [33] and other cancer lines (Supplementary Figure S1C), we adjusted the relative ectopic HMGA2 mRNA below the limit of pathological level (from 100 to 2000 multiples) in different WI38 cells infected with different doses of virus (Figure 1A). Data from Figure 1(B) show that the cell growth was dramatically inhibited depending upon the doses of HMGA2 overexpression, and the 500-multiple relative ectopic HMGA2 mRNA level, within a pathological range, was used in the following experiments. Intriguingly, in addition to the detection of senescence phenotypes (Supplementary Figure S1A) and SAHF (senescence-associated heterochromatin foci)-like foci (Figure 1C, H2-GFP 4d), we observed a consecutive change of the heterochromatin foci containing HMGA2-GFP protein in WI38 cells as monitored by fluorescence microscopy after HMGA2 expression (Figure 1C). Specifically, the SAHF-like foci became enlarged at day 6 (Figure 1C, HMGA2-GFP 6d), and these foci were apparently co-localized with the H3K9me3 (tri-methylated histone H3 at lysine 9) (Supplementary Figure S1B). At day 8 of HMGA2 overexpression, the nuclei became condensed (Figure 1C, H2-GFP 8d), which is a typical change of apoptotic nuclei [34]. Moreover, the apoptotic bodies began to emerge in a number of cells at day 9 (Figure 1C, H2-GFP 9d), along with a decrease in cell number (Figure 1B, H2-GFP 500×).

### HMGA2 overexpression arrested WI38 cells at G2/M

**Figure 2:**
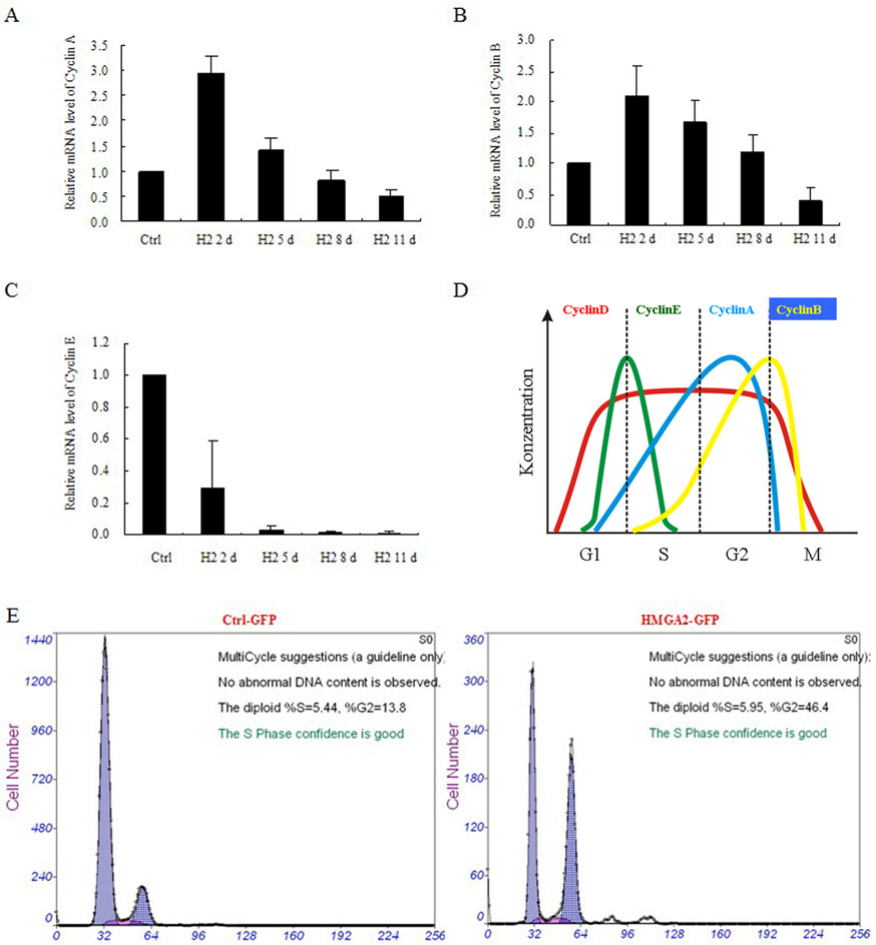
HMGA2-induced G2/M cell cycle arrest in WI38 cells Relative mRNA levels of CyclinA (**A**), CyclinB (**B**) and CyclinE (**C**) in WI38 cells were assessed by qPCR at indicated time points after HMGA2 expression. (**D**) Illustration of changes of cell cycle-related proteins during a normal cell cycle progression. (**E**) WI38 cells expressing HMGA2 at day 5 was analysed by flow cytometry using PI staining, showing that the dramatic increase of percentage (from 13.4 to 46.4%) of cells arrested at G2/M phase.

We further investigated the molecular events during the HMGA2-induced WI38 cell-cycle arrest. The qRT–PCR assays detected the up-regulation of CyclinA and CyclinB mRNAs (Figures 2A and 2B) and down-regulation of CyclinE mRNA (Figure 2C) at day 2 of HMGA2 expression. However, the CyclinA, B and E mRNAs decreased at day 11 post-HMGA2 expression (Figures 2A–2C). These results implicated an increase of cell population at G2/M phase, since CyclinA and B are responsible for G2/M checkpoint transition in cell-cycle progression; whereas CyclinE is responsible for G1/S checkpoint, as illustrated in Figure 2(D). The flow cytometric assay confirmed that WI38 cells expressing HMGA2 were arrested at G2/M phase at day 5 (Figure 2E).

### HMGA2-induced apoptosis in human primary fibroblasts

**Figure 3:**
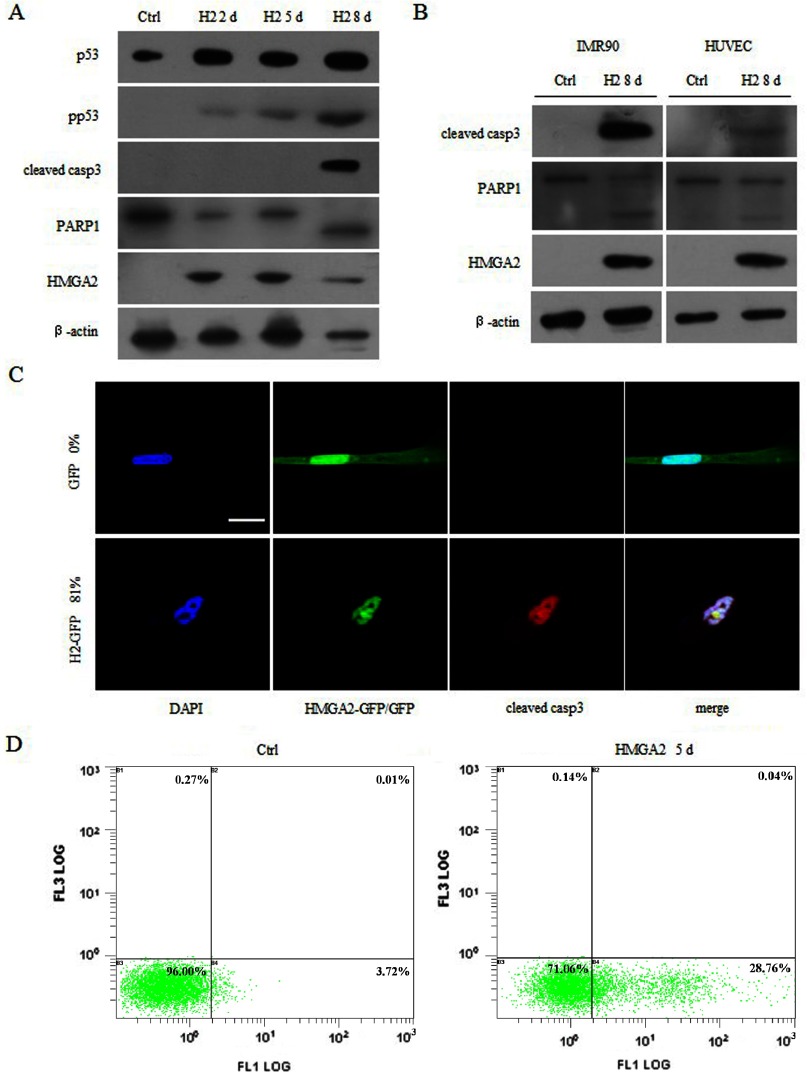
Characterization of apoptosis in human primary fibroblasts expressing HMGA2 (**A**) Up-regulation of the apoptotic proteins p53, pho-p53 and cleaved caspase 3; and degradation of caspase-3 substrate PARP in WI38 cells expressing HMGA2 at indicated time points. (**B**) Cleaved caspase 3 and PARP were detected in IMR90 and HUVEC cells expressing HMGA2 at day 8. (**C**) Immunofluorescence of WI38 cells expressing HMGA2 at day 8, showing the increase of cleaved caspase 3. Scale bar: 20 μm. (**D**) WI38 cells expressing HMGA2 at day 5 were analysed by flow cytometry using Annexin-V and PI staining. The percentage of apoptotic cells, characterized by the positive Annexin-V and the negative PI staining, was dramatically increased from 3.72 to 28.76%.

We next intended to determine whether the HMGA2-induced cell growth arrest was achieved through an apoptotic pathway. We first assessed the expression levels of the apoptotic-related proteins, and we found that the p53, phosphorylated p53 (Figure 3A) and cleaved caspase-3 protein levels were prominently up-regulated upon HMGA2 overexpression (Figures 3A and 3C). Furthermore, the degradation of PARP (Figure 3A), a caspase-3 substrate, was readily evident in WI38 cells expressing HMGA2 in 8 days. Similar results were also obtained in other primary cells, including IMR90 and HUVEC cells (Figure 3B), suggesting that this may be a common feature of the human primary cells. The HMGA2-induced apoptosis was further verified by flow cytometric assays with Annexin-V and PI staining, which revealed that the population of Annexin-V positive cells significantly increased in WI38 cells expressing HMGA2, compared with the control cells (Figure 3D). Only few cells underwent necrosis-like cell death (characterized by PI incorporation) (Figure 3D). These results indicate that overexpression of HMGA2 was sufficient to induce apoptosis in different primary fibroblasts.

### Release of mitochondrial cytochrome *c* and formation of apoptosomes in apoptotic cells expressing HMGA2

**Figure 4:**
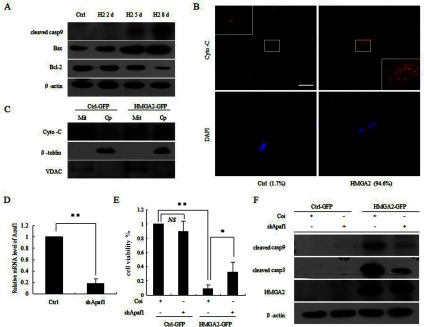
Release of cytochrome *c* from mitochondria was required for HMGA2-induced WI38 cell apoptosis (**A**) The decrease of Bcl-2 levels with a concomitant increase in Bax and cleaved caspase 9, as measured by immunoblotting in apoptotic WI38 cells induced by HMGA2 at indicated time point. (**B**) Immunofluorescence images showing the release of mitochondrial cytochrome *c* in HMGA2-expressing cells at day 5 (enlarged areas in frames). Scale bar: 50 μm. (**C**) Mitochondrial-cytosolic extracts were prepared from cells expressing GFP or HMGA2-GFP at day 5 and analysed for the cytochrome *c* by Western blotting, showing the release of cytochrome *c* from mitochondria in response to HMGA2 overexpression. VDAC as a mitochondrial marker and tubulin as a cytosolic marker were detected. (**D**) Verification of the silencing efficiency of shApaf1 by qPCR in WI38 cells. (**E**) Apaf1 deficient WI38 cells partially escape from HMGA2 induced cell death as calculated in relative survival percentage using MTT assay at day 8. (**F**) Attenuated levels of cleaved caspase 9 and -3 were detected in cells treated as in (**E**).

As shown in Figure 4(A), HMGA2 also triggered the caspase-9 activation preceding the caspase-3 activation, concurrent with the increase of Bax and decrease of Bcl-2 in apoptotic WI38 cells. Furthermore, the release of cytochrome *c*, a crucial step in triggering the formation of the apoptosome and subsequent activation of the effector caspase, was detected by using immunofluorescence and western blotting (Figures 4B and 4C). We next established the WI38 cell line stably expressing shRNAs (small-hairpin RNAs) directed against Apaf1, a cytoplasmic factor that binds with cytochrome *c* and triggers the formation of the apoptosome and the subsequent activation of caspase 9. The silencing efficiency of the Apaf1 shRNA in WI38 cells was confirmed (Figure 4D). We show that when challenged with the HMGA2 overexpression, the Apaf1-deficient cells exhibited remarkable resistance to cell death (Figure 4E). Meanwhile, activation of downstream effector caspases (cleaved caspase 3/9) was detected (Figure 4F). These results indicate that the mitochondrial death pathway was activated and required for HMGA2-induced apoptosis in WI38 cells.

### Caspase 2 was activated and required for HMGA2-induced apoptosis in WI38 cells

**Figure 5:**
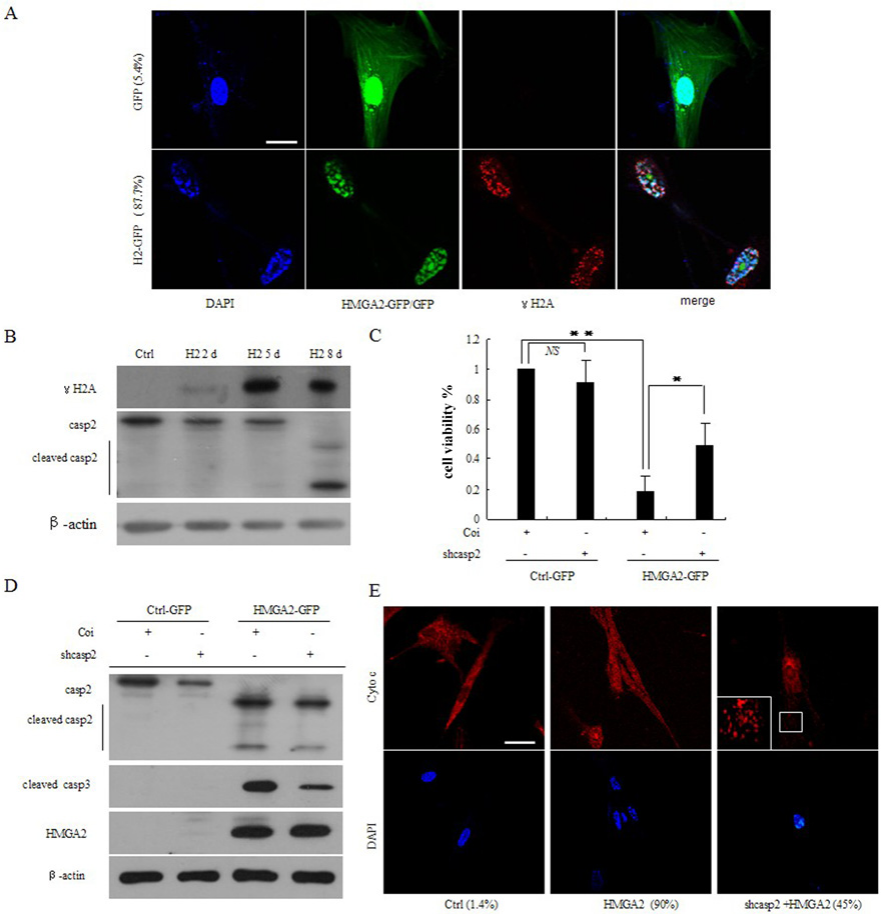
Caspase-2 was crucial for HMGA2-induced apoptosis in WI38 cells (**A**) DNA-damage foci labelled by γH2A in WI38 cells expressing HMGA2 at day 5. The percentage of γH2A positive cells was shown in brackets. Scale bar: 20 μm. (**B**) Western blots showing the increase of γH2A and activated caspase 2 in WI38 cells expressing HMGA2 at indicated time. (**C**) Caspase-2-deficient WI38 cells partially escaped from HMGA2-induced cell death as manifested in relative survival percentages calculated from MTT assay at day 8. (**D**) Attenuated levels of cleaved caspase 2 and -3 in cells treated as in (**C**). (**E**) Immunofluorescence images showing that the cytochrome *c* release from mitochondria induced by HMGA2 was interrupted by shCaspase 2. The percentage of cytochrome *c* diffusion positive cells was shown in brackets. Scale bar: 50 μm.

The observation that the γH2A-DNA damage foci were accumulated in HMGA2-expressing WI38 cells (Figures 5A and 5B) implicates the involvement of DNA damage in HMGA2-induced apoptosis. Since caspase 2 has been shown to play critical roles in stress-induced apoptosis [23,26], we sought to determine whether this caspase was involved in the apoptotic process observed in the present study. Indeed, we found that the HMGA2-expressing apoptotic WI38 cells displayed a marked increase in activated caspase 2 (Figure 5B, H2 8d). Similar results were also obtained when the IMR90 and HUVEC cells were infected with lentivirus containing HMGA2-GFP at day 8 (results not shown). Furthermore, WI38 cells transfected with Caspase-2 siRNA exhibited significantly lower cell death rates and weaker activation of effector caspases compared with the control (Figures 5C and 5D), suggesting that caspase 2 was necessary for the apoptotic pathway induced by HMGA2. To determine whether caspase 2 regulates cytochrome *c* release, WI38 cells were treated with the shRNA targeting caspase 2 before HMGA2 overexpression, and cytochrome *c* release was subsequently assessed by immunofluorescence microscopy. The results showed that in the presence of shcaspase 2, cytochrome *c* release was partially blocked in HMGA2 expressing cells (Figure 5E). Taken together, these data implicate that caspase 2 contributed to the HMGA2-induced apoptosis in primary cells.

## DISCUSSION

Over the past few years, intense research has shown that HMGAs are involved in many cellular processes including proliferation, differentiation and neoplasm [35]. In the present study, we demonstrate a previously unidentified function of HMGA2 protein to induce cellular apoptosis in primary cells. The observed HMGA2-induced apoptosis is modulated through a mechanism apparently distinct from that identified in an earlier study, which showed that HMGA1, the other member of HMGA family, induced apoptosis through up-regulation of CyclinA [36]. In the present study, we detected the apoptotic processes in different primary cells that overexpressed high level of HMGA2 (Figures 3A and 3B), and the model we used was similar to that in previous studies with HMGA2-induced senescence [8,37]. Apparently, HMGA2 is capable of either triggering an apoptosis or a senescence process, as manifested in this and other studies, respectively. We figure that the contradictory results may probably be ascribed to the different expression levels of HMGA2 in the cells (Figure 1B). Interestingly, we found that WI38 cells ectopically expressing a relative HMGA2 mRNA level higher than 500-multiple embarked upon the apoptotic process (Figure 1B, H2-GFP 500×), whereas cells expressing only a 100-multiple relative HMGA2 mRNA level tended to undergo a senescence process (Figure 1B, H2-GFP 100×). The similar phenomenon was also seen in Narita's study [8], in which the authors used retroviruses to express HMGA fused to GFP, driven by either the strong CMV (cytomegalovirus) promoter or by the weaker LTR promoter, leading to different levels of transgene expression. They found that cells expressing high levels of HMGA1/2 protein underwent an acute cell cycle arrest resulted in decrease of cell population. In contrast, low expression level of HMGA1/2 did not cause severe growth arrest, instead, these cells exhibited an early replicative exhaustion; however, the relative ectopically expressing HMGA1/2 mRNA levels were not determined in Narita's study [8]. Noticeably, in our study, the occurrence of senescence phenotypes, including the increase of SA-β-galactosidase activity and formation of the SAHF, were also detected in cells undergoing apoptosis (Supplementary Figure S1). Interestingly, the decreased mRNA level of Wnt2 was also detected in our cellular model (Supplementary Figure S2). This phenomenon consists with the results in a previous report that in tumour cells, Wnt2 was one of the genes with over 2-fold down-regulation by HMGA2 [38]. We postulate that the down-regulation of Wnt pathway upon HMGA2 overexpression in primary cells may be the reason for HMGA2-induced SAHF formation and senescence phenotypes, as described by Narita et al. [8]. Similarly, a number of other studies also found that down-regulation of Wnt2 initiated the SAHF formation [39,40]. Nevertheless, other mechanisms may also be involved in HMGA2-mediated cell growth arrest. Our data may provide an explanation of why the senescence phenotypes and the SAHF-like foci were detected in this apoptosis model.

Although the partial senescence phenotypes can be observed in the experimental model system used in the present study, our results support the notion that the apoptotic pathway is the major contribution to the HMGA2-induced cell growth arrest in human primary cells. Moreover, we identified the apparent accumulation of DNA damage in HMGA2 expressing cells, which is consistent with the previous study [9]. Presumably, the HMGA2-induced DNA damage may initialize the caspase-2 activation and further active the downstream effecter caspases through MOMP. Although the detailed mechanisms about how DNA damage activated caspase-2 and cytochrome *c* release in our system remain to be further explored, our data strongly suggest that caspase-2 activation is a crucial process in HMGA2-induced apoptosis. Probably, this process represents a native defence machinery to avoid aberrant cellular proliferation and to eliminate the accumulation of genetic defects in oncoprotein HMGA2-overexpressing primary cells. Deregulation of this apoptotic pathway may confer the cancer cells with resistance to cell death even under a severe DNA damage stress [41]. Additionally, caspase 2 has been attracting a great deal of research attention since its activation was found to induce apoptosis in many tumour cells [42]. Thus, the tumour suppressor function of caspase 2 may become a new option in therapeutic strategy aimed at control of tumour growth under a high level expression of HMGA2.

**Figure 6:**
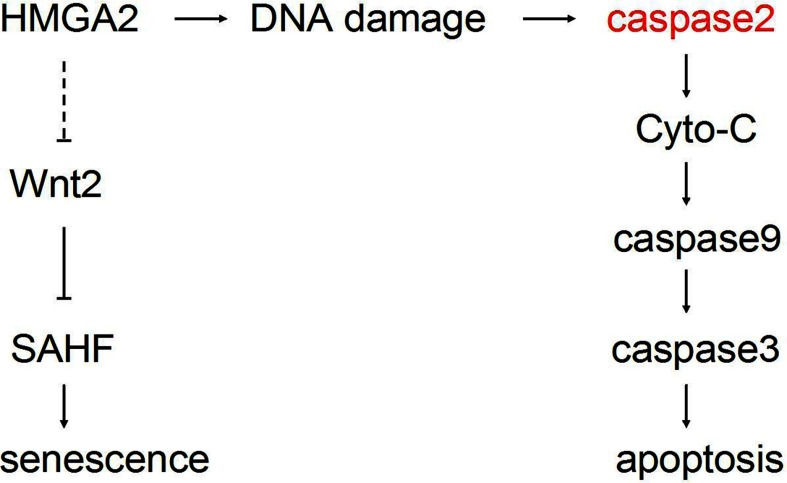
A diagram of possible pathways that control the HMGA2-induced cell growth arrest in primary cells

To summarize, the possible signalling pathways that are involved in HMGA2-mediated cell growth arrest either through senescence or through apoptosis in primary cells has been diagrammatically illustrated in Figure 6. Briefly, high-level expression of HMGA2 induces cell growth arrest mainly depending on the apoptosis process, which is activated by accumulation of DNA damage that may initialize caspase-2 activation and further induces MOMP to active downstream caspases 9 and -3. In addition, we propose that down-regulation of Wnt2 may be the reason for HMGA2-triggered SAHF assembly and senescence phenotypes in our model.

Overall, based on data both from the present study and from others, we propose that both senescence and apoptosis contribute to anti-proliferative function of HMGA2 proteins in primary cells.

## Online data

Supplementary data

## References

[1] Manfioletti G., Giancotti V., Bandiera A., Buratti E., Sautiere P., Cary P., Crane-Robinson C., Coles B., Goodwin G. H. (1991). cDNA cloning of the HMGI-C phosphoprotein, a nuclear protein associated with neoplastic and undifferentiated phenotypes. Nucleic Acids Res..

[2] Johnson K. R., Lehn D. A., Reeves R. (1989). Alternative processing of mRNAs encoding mammalian chromosomal high-mobility-group proteins HMG-I and HMG-Y. Mol. Cell. Biol..

[3] Chiappetta G., Avantaggiato V., Visconti R., Fedele M., Battista S., Trapasso F., Merciai B. M., Fidanza V., Giancotti V., Santoro M., Simeone A., Fusco A. (1996). High level expression of the HMGI (Y) gene during embryonic development. Oncogene.

[4] Hirning-Folz U., Wilda M., Rippe V., Bullerdiek J., Hameister H. (1998). The expression pattern of the Hmgic gene during development. Genes Chrom. Cancer..

[5] Gattas G. J., Quade B. J., Nowak R. A., Morton C. C. (1999). HMGIC expression in human adult and fetal tissues and in uterine leiomyomata. Genes Chrom. Cancer.

[6] Fusco A., Fedele M. (2007). Roles of HMGA proteins in cancer. Nat. Rev. Cancer.

[7] Li A. Y., Lin H. H., Kuo C. Y., Shih H. M., Wang C. C., Yen Y., Ann D. K. (2011). High-mobility group A2 protein modulates hTERT transcription to promote tumorigenesis. Mol. Cell Biol..

[8] Narita M., Narita M., Krizhanovsky V., Nunez S., Chicas A., Hearn S. A., Myers M. P., Lowe S. W. (2006). A novel role for high-mobility group a proteins in cellular senescence and heterochromatin formation. Cell.

[9] Li A. Y., Boo L. M., Wang S. Y., Lin H. H., Wang C. C., Yen Y., Chen B. P., Chen D. J., Ann D. K. (2009). Suppression of nonhomologous end joining repair by overexpression of HMGA2. Cancer Res..

[10] Otsuki Y., Li Z., Shibata M. A. (2003). Apoptotic detection methods–from morphology to gene. Prog. Histochem. Cytochem..

[11] Kroemer G., Galluzzi L., Vandenabeele P., Abrams J., Alnemri E. S., Baehrecke E. H., Blagosklonny M. V., El-Deiry W. S., Golstein P., Green D. R. (2009). Classification of cell death: recommendations of the Nomenclature Committee on Cell Death 2009. Cell Death Differ..

[12] Meier P., Vousden K. H. (2007). Lucifer's labyrinth–ten years of path finding in cell death. Mol. Cell.

[13] Thornberry N. A., Lazebnik Y. (1998). Caspases: enemies within. Science.

[14] Shi Y. (2004). Caspase activation: revisiting the induced proximity model. Cell.

[15] Slee E. A., Harte M. T., Kluck R. M., Wolf B. B., Casiano C. A., Newmeyer D. D., Wang H. G., Reed J. C., Nicholson D. W., Alnemri E. S. (1999). Ordering the cytochrome c-initiated caspase cascade: hierarchical activation of caspases-2, -3, -6, -7, -8, and -10 in a caspase-9-dependent manner. J. Cell Biol..

[16] Li P., Nijhawan D., Budihardjo I., Srinivasula S. M., Ahmad M., Alnemri E. S., Wang X. (1997). Cytochrome c and dATP-dependent formation of Apaf-1/caspase-9 complex initiates an apoptotic protease cascade. Cell.

[17] Boatright K. M., Renatus M., Scott F. L., Sperandio S., Shin H., Pedersen I. M., Ricci J. E., Edris W. A., Sutherlin D. P., Green D. R., Salvesen G. S. (2003). A unified model for apical caspase activation. Mol. Cell.

[18] Scaffidi C., Fulda S., Srinivasan A., Friesen C., Li F., Tomaselli K. J., Debatin K. M., Krammer P. H., Peter M. E. (1998). Two CD95 (APO-1/Fas) signaling pathways. EMBO J..

[19] Jin Z., El-Deiry W. S. (2005). Overview of cell death signaling pathways. Cancer Biol. Ther..

[20] Lamkanfi M., Declercq W., Kalai M., Saelens X., Vandenabeele P. (2002). Alice in caspase land. A phylogenetic analysis of caspases from worm to man. Cell Death Differ..

[21] Troy C. M., Shelanski M. L. (2003). Caspase-2 redux. Cell Death Differ..

[22] Tinel A., Janssens S., Lippens S., Cuenin S., Logette E., Jaccard B., Quadroni M., Tschopp J. (2007). Autoproteolysis of PIDD marks the bifurcation between pro-death caspase-2 and pro-survival NF-kappaB pathway. EMBO J..

[23] Lassus P., Opitz-Araya X., Lazebnik Y. (2002). Requirement for caspase-2 in stress-induced apoptosis before mitochondrial permeabilization. Science.

[24] Baptiste-Okoh N., Barsotti A. M., Prives C. (2008). A role for caspase 2 and PIDD in the process of p53-mediated apoptosis. Proc. Natl. Acad. Sci. U. S. A..

[25] Guo Y., Srinivasula S. M., Druilhe A., Fernandes-Alnemri T., Alnemri E. S. (2002). Caspase-2 induces apoptosis by releasing proapoptotic proteins from mitochondria. J. Biol. Chem..

[26] Robertson J. D., Enoksson M., Suomela M., Zhivotovsky B., Orrenius S. (2002). Caspase-2 acts upstream of mitochondria to promote cytochrome c release during etoposide-induced apoptosis. J. Biol. Chem..

[27] Kumar S. (2009). Caspase 2 in apoptosis, the DNA damage response and tumour suppression: enigma no more?. Nat. Rev. Cancer.

[28] Kitevska T., Spencer D. M., Hawkins C. J. (2009). Caspase-2: controversial killer or checkpoint controller?. Apoptosis.

[29] Zhivotovsky B., Orrenius S. (2005). Caspase-2 function in response to DNA damage. Biochem. Biophys. Res. Commun..

[30] Krumschnabel G., Manzl C., Villunger A. (2009). Caspase-2: killer, savior and safeguard-emerging versatile roles for an ill-defined caspase. Oncogene..

[31] Bouchier-Hayes L., Green D. R. (2012). Caspase-2: the orphan caspase. Cell Death Differ..

[32] Ren K., Lu J., Porollo A., Du C. (2012). Tumor-suppressing function of caspase-2 requires catalytic site Cys-320 and site Ser-139 in mice. J. Biol. Chem..

[33] Di Cello F., Hillion J., Hristov A., Wood L. J., Mukherjee M., Schuldenfrei A., Kowalski J., Bhattacharya R., Ashfaq R., Resar L. M. (2008). HMGA2 participates in transformation in human lung cancer. Mol. Cancer Res..

[34] Wyllie A. H., Beattie G. J., Hargreaves A. D. (1981). Chromatin changes in apoptosis. Histochem. J..

[35] Reeves R. (2001). Molecular biology of HMGA proteins: hubs of nuclear function. Gene.

[36] Fedele M., Pierantoni G. M., Berlingieri M. T., Battista S., Baldassarre G., Munshi N., Dentice M., Thanos D., Santoro M., Viglietto G., Fusco A. (2001). Overexpression of proteins HMGA1 induces cell cycle deregulation and apoptosis in normal rat thyroid cells. Cancer Res..

[37] Shi X., Tian B., Liu L., Gao Y., Ma C., Mwichie N., Ma W., Han L., Huang B., Lu J., Zhang Y. (2013). Rb protein is essential to the senescence-associated heterochromatic foci formation induced by HMGA2 in primary WI38 cells. J. Genet. Genomics.

[38] Wu J., Liu Z., Shao C., Gong Y., Hernando E., Lee P., Narita M., Muller W., Liu J., Wei J. J. (2011). HMGA2 overexpression-induced ovarian surface epithelial transformation is mediated through regulation of EMT genes. Cancer Res..

[39] Banumathy G., Somaiah N., Zhang R., Tang Y., Hoffmann J., Andrake M., Ceulemans H., Schultz D., Marmorstein R., Adams P. D. (2009). Human UBN1 is an ortholog of yeast Hpc2p and has an essential role in the HIRA/ASF1a chromatin-remodeling pathway in senescent cells. Mol. Cell Biol..

[40] Ye X., Zerlanko B., Kennedy A., Banumathy G., Zhang R., Adams P. D. (2007). Downregulation of Wnt signaling is a trigger for formation of facultative heterochromatin and onset of cell senescence in primary human cells. Mol. Cell.

[41] Ho L. H., Taylor R., Dorstyn L., Cakouros D., Bouillet P., Kumar S. (2009). A tumor suppressor function for caspase-2. Proc. Natl. Acad. Sci. U. S. A..

[42] Kim S. H., Dass C. R. (2012). Induction of caspase-2 activation by a DNA enzyme evokes tumor cell apoptosis. DNA Cell Biol..

[43] Wang X., Pan L., Feng Y., Wang Y., Han Q., Han L., Han S., Guo J., Huang B., Lu J. (2008). P300 plays a role in p16(INK4a) expression and cell cycle arrest. Oncogene.

