# Supplementary material for: A novel anti-proliferative role of HMGA2 in induction of apoptosis through caspase 2 in primary human fibroblast cells
Source: Biosci Rep. 2015 Jan 14;35(1):e00169. doi: 10.1042/BSR20140112 (PMC4293904; doi:10.1042/BSR20140112)
Supplement: Supplementary data [file bsr035e169ntsadd.pdf]

Supplementary

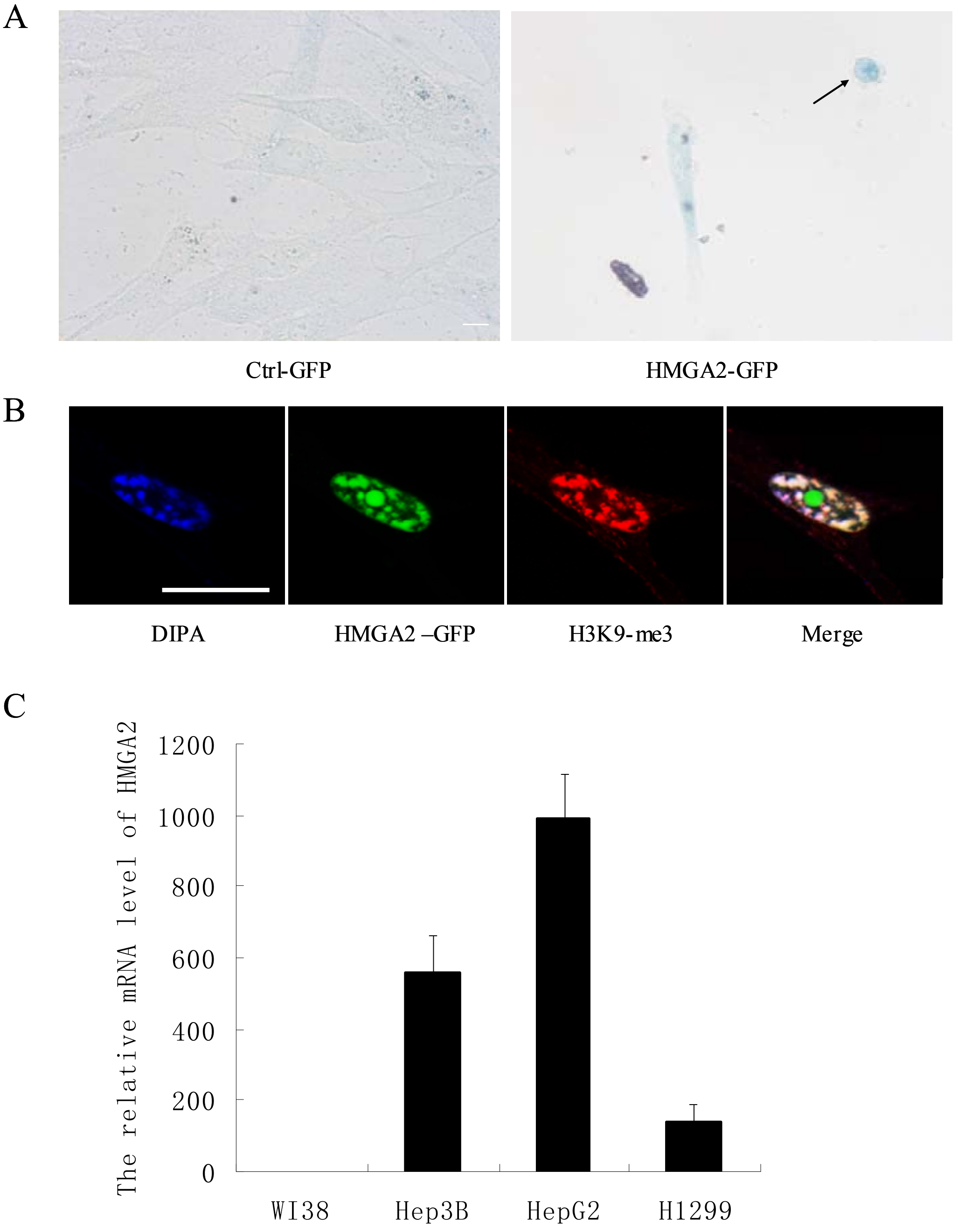

Figure S1. Senescence phenotypes were detected in WI38 cells expressing HMGA2. A. Increased SA-β-galactosidase activity in WI38 cells 8d after HMGA2-GFP overexpression. The arrow denotes the cell with both senescence and apoptotic phenotypes. **B**. Confocal immunofluorescent images of co-localization of chromatin foci with the SAHF marker H3K9me3

in WI38 cells 5d after the overexpression of HMGA2-GFP. Scale bar: 20μm. **C**. The expression level of HMGA2 in different tumor cell lines detected by qPCR

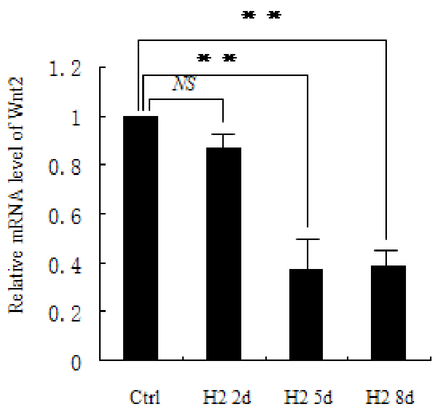

Figure S2. Downregulation of Wnt2 detected by Q-PCR in WI38 cells expressing HMGA2 in indicated times.
